# Experimental infection of dromedaries with Middle East respiratory syndrome-Coronavirus is accompanied by massive ciliary loss and depletion of the cell surface receptor dipeptidyl peptidase 4

**DOI:** 10.1038/s41598-018-28109-2

**Published:** 2018-06-27

**Authors:** Ann-Kathrin Haverkamp, Annika Lehmbecker, Ingo Spitzbarth, Widagdo Widagdo, Bart L. Haagmans, Joaquim Segalés, Julia Vergara-Alert, Albert Bensaid, Judith M. A. van den Brand, Albert D. M. E. Osterhaus, Wolfgang Baumgärtner

**Affiliations:** 1 Department of Pathology, University of Veterinary Medicine Hannover Foundation, 30559 Hannover, Germany; 2 Center for Systems Neuroscience, 30559 Hannover, Germany; 3 Department of Viroscience, Erasmus Medical Center, 3015 CN Rotterdam, The Netherlands; 4 IRTA, Centre de Recerca en Sanitat Animal (CReSA, IRTA-UAB), Campus de la Universitat Autònoma de Barcelona, 08193 Bellaterra, Spain; 5 Departament de Sanitat i Anatomia Animals, Facultat de Veterinària, UAB, 08193 Bellaterra, Barcelona Spain; 6 Department of Pathobiology, Faculty of Veterinary Science, Utrecht University, 3512 JE Utrecht, The Netherlands; 7 Research Center for Emerging Infections and Zoonoses (RIZ), University of Veterinary Medicine Hannover Foundation, 30559 Hannover, Germany

## Abstract

Middle East respiratory syndrome (MERS) represents an important respiratory disease accompanied by lethal outcome in one-third of human patients. Recent data indicate that dromedaries represent an important source of infection, although information regarding viral cell tropism and pathogenesis is sparse. In the current study, tissues of eight dromedaries receiving inoculation of MERS-Coronavirus (MERS-CoV) after recombinant Modified-Vaccinia-Virus-Ankara (MVA-S)-vaccination (n = 4), MVA-vaccination (mock vaccination, n = 2) and PBS application (mock vaccination, n = 2), respectively, were investigated. Tissues were analyzed by histology, immunohistochemistry, immunofluorescence, and scanning electron microscopy. MERS-CoV infection in mock-vaccinated dromedaries revealed high numbers of MERS-CoV-nucleocapsid positive cells, T cells, and macrophages within nasal turbinates and trachea at day four post infection. Double immunolabeling demonstrated cytokeratin (CK) 18 expressing epithelial cells to be the prevailing target cell of MERS-CoV, while CK5/6 and CK14 expressing cells did not co-localize with virus. In addition, virus was occasionally detected in macrophages. The acute disease was further accompanied by ciliary loss along with a lack of dipeptidyl peptidase 4 (DPP4), known to mediate virus entry. DPP4 was mainly expressed by human lymphocytes and dromedary monocytes, but overall the expression level was lower in dromedaries. The present study underlines significant species-specific manifestations of MERS and highlights ciliary loss as an important finding in dromedaries. The obtained results promote a better understanding of coronavirus infections, which pose major health challenges.

## Introduction

In June 2012 a novel lineage C betacoronavirus (HCoV-EMC) was identified in a patient from the Kingdom of Saudi Arabia who suffered from acute pneumonia and renal failure^1^. Subsequently, the virus was named Middle East respiratory syndrome coronavirus (MERS-CoV) in accordance with the geographical area of its first description and main occurrence^2^. Until today, MERS-CoV represents an existential threat to global health since the virus spread to 27 countries and caused more than 2000 laboratory confirmed cases in humans including 730 fatal cases, which equals approximately one third of all affected patients (World Health Organization (2017) Middle East respiratory syndrome coronavirus, available at http://www.who.int/emergencies/mers-cov/en/, accessed October 27, 2017).

The sequence of MERS-CoV was determined to be closely related to other betacoronaviruses isolated from bats and therefore a bat origin has been proposed early after genomic characterization^3–8^. However, transmission of MERS-CoV to humans was suspected to occur *via* an intermediate mammalian host, since the majority of human Middle East respiratory syndrome (MERS) patients did not state any direct contact to bats prior to disease onset^6,9^. Similarly, severe acute respiratory syndrome coronavirus (SARS-CoV), a betacoronavirus of the lineage B, originated from bats^10^ and spread from palm civets to humans in 2002/2003^11^.

In 2013, one year after the initial description of MERS, serological investigations in livestock species suspected dromedaries (*Camelus dromedarius*) to act as potential intermediate hosts for MERS-CoV^12^. Subsequent research on index cases^13^, serological sampling^14^, and virus isolation^15^ revealed a transmission from dromedaries to humans and confirmed dromedaries as the major reservoir for human infections^16^. Consequently, MERS-CoV infection in dromedaries was documented by serological surveys in large parts of the Middle East, the Canary Islands, and Africa, as previously summarized by Hemida *et al*.^17^ and additionally in certain parts of Southern Asia^18^ and Western Africa^19^. Recently, MERS-CoV has also been detected in alpacas from Qatar^20^, but not in any other domestic livestock species^14^.

In recent animal trials with dromedaries, experimental MERS-CoV infection led to mild transient upper respiratory tract disease characterized by mild to moderate rhinitis with nasal discharge, tracheitis, and bronchitis accompanied by shedding of large quantities of virus from the upper respiratory tract. Viral antigen was additionally detected in regional lymph nodes but not in any other extra-respiratory organ^21,22^. The serine exopeptidase dipeptidyl peptidase 4 (DPP4; also known as CD26) has been identified as a major virus receptor in both humans and dromedaries^23,24^ and is expressed in the lower respiratory tract of humans and the upper respiratory tract of dromedaries^25^. More recently, carcinoembryonic antigen-related cell adhesion molecule 5 (CEACAM5) has been described as an additional cellular binding target for MERS-CoV that may facilitates virus entry^26^. Additionally, binding of MERS-CoV to sialic acids has been demonstrated in a newly published study by Li and colleagues and is suggested to represent another important factor in viral host tropism^27^.

Despite two recent animal trials^21,22^ and the successful establishment of an orthopoxvirus-based vaccine against MERS-CoV in dromedaries^22^, the virus infection triggered immune response and the exact cell tropism have not been evaluated so far, since valuable markers for immune cell phenotyping in dromedaries were hardly established. In order to facilitate such studies and other investigations in camels, a panel of antibodies for identification of different immune cell subsets has been recently tested^28^. Since dromedaries play a key role in transmitting MERS-CoV to humans, it was the aim of the present study to describe MERS-CoV associated lesions in the nasal epithelium and viral cell tropism of experimentally infected dromedaries in closer detail.

## Results

### Experimental MERS-CoV infection of dromedaries leads to high virus load in nasal turbinates and trachea accompanied by necro-suppurative inflammation at day 4 post infection

**Figure 1:**
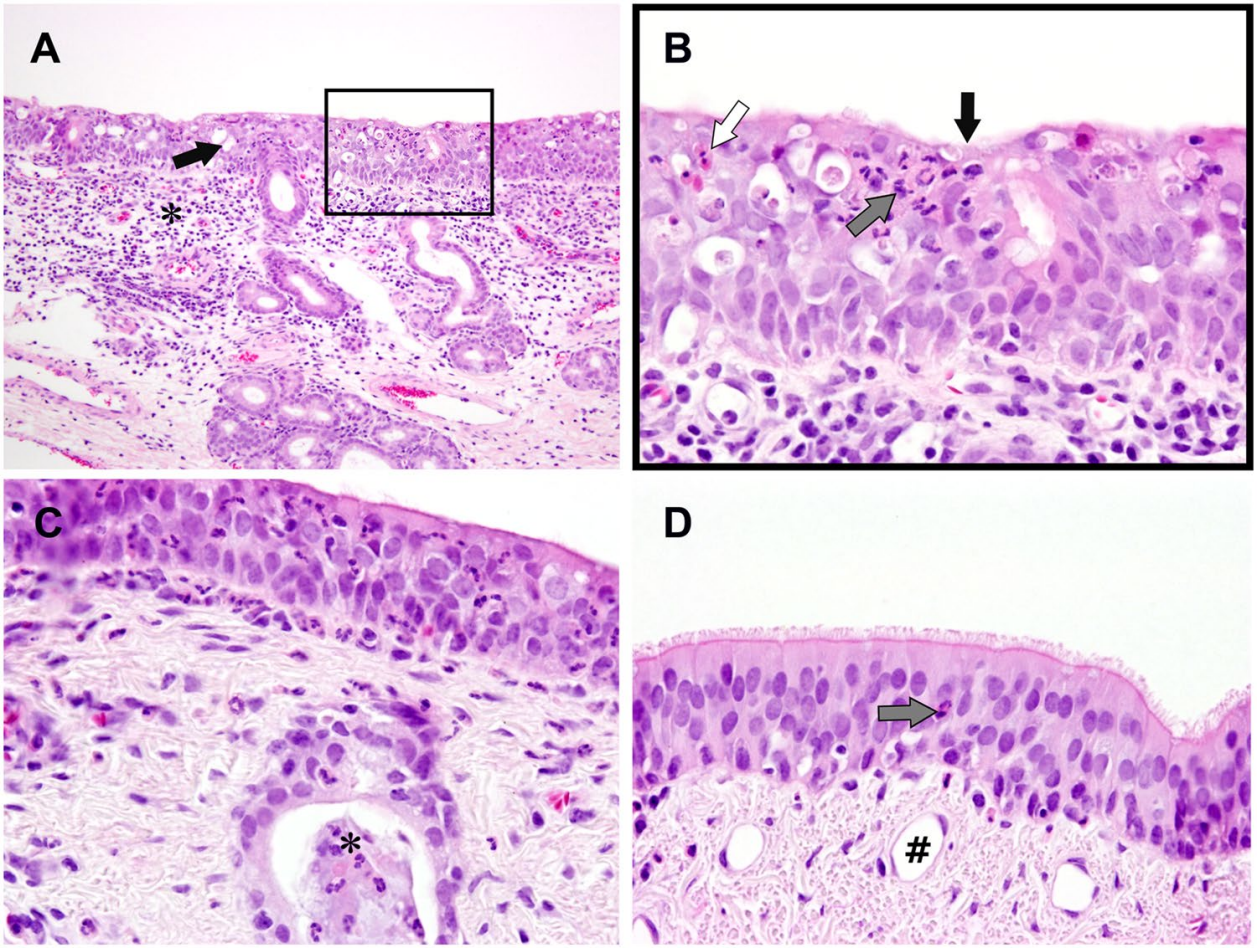
Histological findings in nasal turbinates, trachea, and bronchus of a MERS-Coronavirus (MERS-CoV)-infected, mock-vaccinated dromedary. (**A**) Respiratory epithelium of the nasal turbinates. Multifocal cavity formation within the epithelium (black arrow) and infiltration of lamina propria and submucosa by moderate numbers of lymphocytes, macrophages and neutrophilic granulocytes (asterisk), 100x. (**B**) Respiratory epithelium of the nasal turbinates. Higher magnification of the inset in (**A**). Erosion (black arrow), exocytosis of neutrophilic granulocytes (grey arrow) and detection of single apoptotic epithelial cells within the epithelium (white arrow), 400x. (**C**) Trachea. Exocytosis of neutrophilic granulocytes, infiltration of lamina propria and submucosa by lymphocytes, neutrophilic granulocytes and macrophages and accumulation of neutrophilic granulocytes and cellular debris within the lumen of a submucosal gland (asterisk), 400x. (**D**) Bronchus. Mild exocytosis of neutrophilic granulocytes (grey arrow) and dilation of lymphatics in the submucosa (rhombus), 400x. A-D: HE staining.

Evaluation of hematoxylin and eosin (HE) stained sections taken from respiratory epithelium of the nasal turbinates of mock-vaccinated animals at 4 days post infection (dpi) revealed mild to moderate, segmental hyperplasia of the epithelium. Moderate exocytosis of neutrophilic granulocytes, apoptosis and single cell necrosis of epithelial cells were observed within all layers of the epithelium and frequently accompanied by formation of small cavities in the apical epithelium (Fig. 1A). Additionally, a partial loss of apical epithelial cells (erosion) was occasionally present within certain areas with marked inflammation (Fig. 1B). The epithelial surface was covered multifocally by large amounts of mucus, as well as viable and degenerated neutrophilic granulocytes and cellular debris. Lamina propria and submucosa displayed mild to moderate edema and infiltration of moderate numbers of lymphocytes and macrophages as well as single neutrophilic granulocytes, which were predominantly located next to blood vessels. Histology of trachea and bronchi revealed only mild lesions which were characterized by mild exocytosis of neutrophilic granulocytes and mild, segmental infiltration of lamina propria and submucosa by lymphocytes, macrophages, and low numbers of neutrophilic granulocytes (Fig. 1C,D). Rarely epithelial erosion and accumulations of degenerated neutrophilic granulocytes within the apical epithelial layers (pustules) of the trachea were detected. The MVA-S-vaccinated animals showed similar but less severe lesions at 4 dpi in nasal turbinates, trachea, and bronchi which were rarely accompanied by degeneration, loss, and necrosis of single epithelial cells in the nasal turbinates. At 14 dpi, very mild lesions were detectable in nasal turbinates, trachea, and bronchi of all investigated animals (MVA-S-vaccinated and mock-vaccinated) characterized by mild, multifocal, lymphoplasmahistiocytic and neutrophilic infiltration of lamina propria and submucosa and rare exocytosis of neutrophilic granulocytes (data not shown). Rarely, follicular aggregates of lymphoid cells were detectable in the lamina propria and submucosa of nasal turbinates and trachea in both groups and at all investigated time points. Within pulmonary, tracheal and cervical lymph nodes as well as within tonsils mild to moderate follicular hyperplasia and single apoptotic lymphocytes were present. All other non-respiratory organs and the lungs did not show any significant lesions.

**Figure 2:**
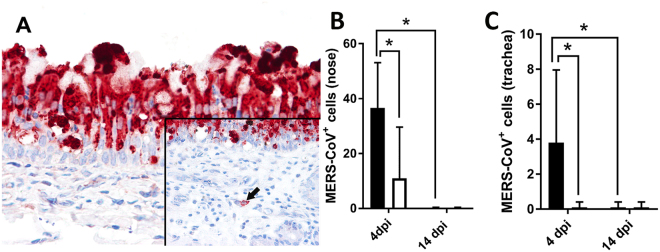
Expression of viral antigen in the nasal turbinates of MERS-Coronavirus (MERS-CoV)-infected, mock-vaccinated and MVA-S-vaccinated dromedaries. (**A**) Nasal respiratory epithelium of a mock-vaccinated dromedary. Abundant antigen in the cytoplasm of apical epithelial cells, 200x. Insert: Detection of antigen in single round cells in the submucosa (black arrow). Positive staining cells are characterized by moderate amounts of cytoplasm and bean-shaped nuclei, 400x. (**B**) Quantification of virus antigen in the nasal turbinates. Significantly decreased virus load in MVA-S-vaccinated animals (white columns) compared to mock-vaccinated animals (black columns) at 4 days post infection (dpi). Significant decrease of virus antigen in mock-vaccinated animals between 4 and 14 dpi. (**C**) Quantification of virus antigen in the trachea. Significantly lowered virus load in MVA-S-vaccinated animals compared to mock-vaccinated animals at 4 dpi. Significant decrease of virus antigen in mock-vaccinated animals in the following ten days. A, MERS-CoV nucleocapsid-specific immunohistochemistry, AEC. B,C: column bars, Mann Whitney-U-Test, *p < 0.05.

In line with these observations, high amounts of MERS-CoV antigen were detected within the respiratory epithelium of the nasal turbinates of mock-vaccinated dromedaries at 4 dpi by immunohistochemistry in areas with most severe lesions (Fig. 2A). Rarely, MERS-CoV antigen was also present within single round cells located in the submucosa of the nasal turbinates, which were presumed to represent macrophages based on histological characteristics (Fig. 2A, insert). In addition, single positive cells were detected in the epithelium of the trachea in both mock-vaccinated animals and in the epithelium of a large bronchus of one mock-vaccinated animal at 4 dpi. In MVA-S-vaccinated animals low numbers of positive staining cells were present in the epithelium of the nasal turbinates at 4 dpi. No virus was detectable in trachea and bronchi of these dromedaries. At 14 dpi, virus antigen was not detectable in the upper respiratory tract of MVA-S-vaccinated animals but very rarely within the nose of mock-vaccinated animals (Fig. 2B,C). All these findings are in line with previous studies by the co-authors^22^ and other investigators^21^.

### MERS-CoV infection in dromedaries is accompanied by a strong but transient infiltration of T cells and macrophages

**Figure 3:**
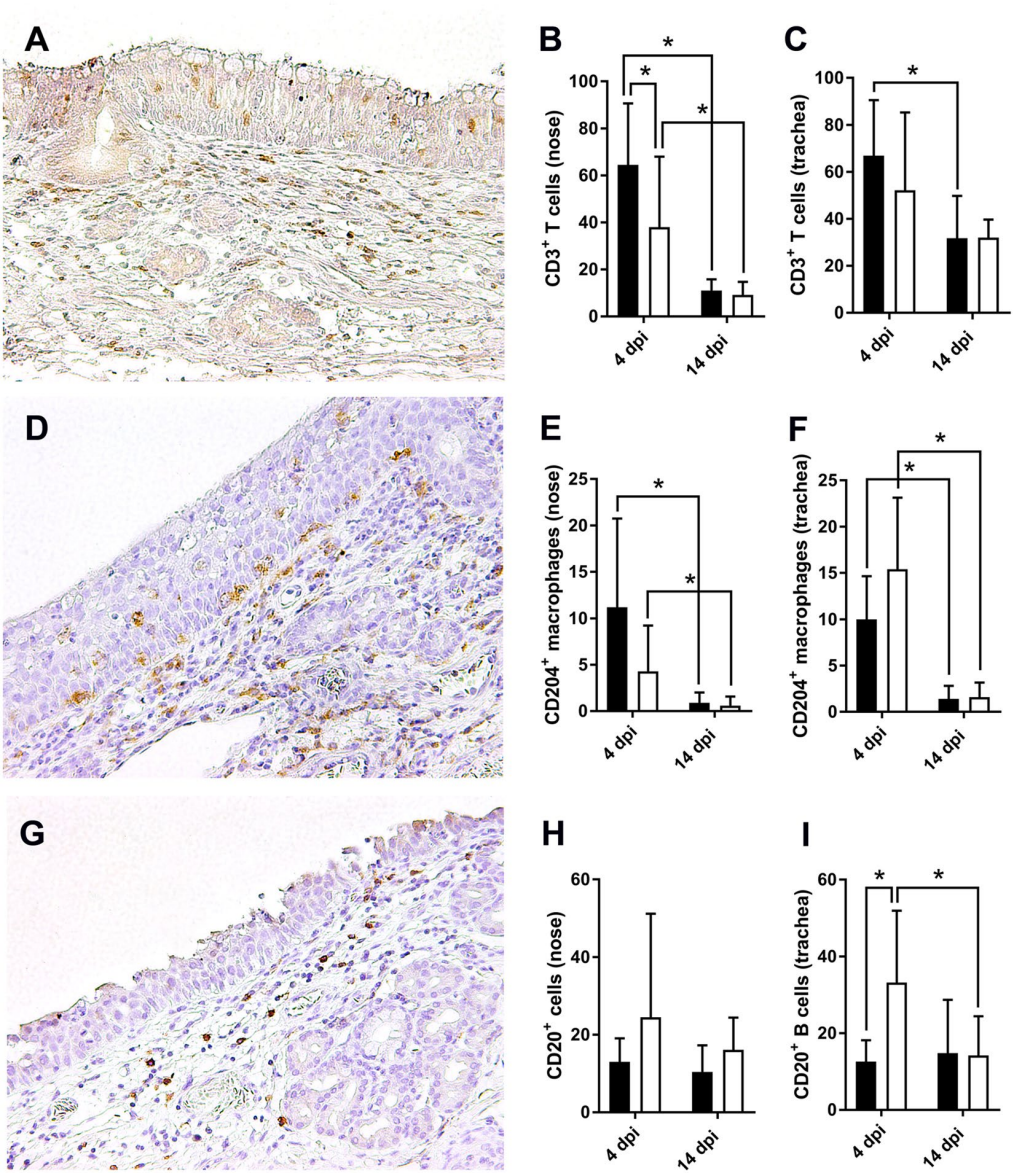
Immunophenotyping of inflammatory cells in nasal turbinates and trachea of MERS-Coronavirus (MERS-CoV)-infected, MVA-S-vaccinated and MERS-CoV-infected, mock-vaccinated dromedaries. (**A**) Respiratory epithelium of the nasal turbinates. High numbers of CD3^+^ T cells are detectable within lamina propria, submucosa and epithelium, 200x. (**B**) Quantification of T cells in the nasal turbinates. Significant decrease of T cells in MVA-S-vaccinated animals (white columns) compared to mock-vaccinated animals (black columns) at 4 days post infection (dpi). Significant decrease of T cells in both groups within the following ten days. (**C**) Quantification of CD3^+^ T cells in the trachea. No differences between MVA-S-vaccinated (white columns) and mock-vaccinated (black columns) dromedaries at both investigated time points. Significant decrease of T cells in mock-vaccinated animals between 4 and 14 dpi. (**D**) Respiratory epithelium of the nose. CD204^+^ macrophages are predominantly located in lamina propria and submucosa, 200x. (**E**) Quantification of macrophages in nasal turbinates and trachea (**F**). No differences between MVA-S-vaccinated (white columns) and mock-vaccinated (black columns) dromedaries at both investigated time points in both organs. Significant decrease of macrophages in both groups and organs over time. (**G**) Respiratory epithelium of the nasal turbinates. CD20^+^ B cells are present in lamina propria and submucosa, 200x. (**H**) Similar numbers of B cells in MVA-S-vaccinated (white columns) and mock-vaccinated (black columns) animals at both time points. (**I**) Significant increase of B cells in MVA-S-vaccinated animals (white columns) compared to mock-vaccinated animals (black columns) at 4 dpi. Significant decrease of B cells in the following ten days in MVA-S-vaccinated animals. A: CD3-specific immunohistochemistry, DAB, D: CD204-specific immunohistochemistry, DAB, G: CD20-specific immunohistochemistry, DAB, B, C, E, F, H, I: column bars, Mann Whitney-U-Test, *p < 0.05.

Since high amounts of virus antigen were present within the nasal turbinates, and to a lesser extent also within the trachea of mock-vaccinated dromedaries at 4 dpi, these tissues were further analyzed by additional immunohistochemistry using different antibodies. Phenotyping of inflammatory cells revealed high numbers of CD3^+^ T cells in lamina propria and submucosa of nasal turbinates and trachea in mock-vaccinated dromedaries at 4 dpi (Fig. 3A–C). Comparison of mock-vaccinated and MVA-S-vaccinated animals at that time point revealed significantly increased numbers of T cells within the nasal turbinates, but not within the trachea of mock-vaccinated animals. At 14 dpi, the numbers of T cells were scarce. Similarly, increased numbers of CD204^+^ macrophages were detected at 4 dpi compared to 14 dpi in nasal turbinates and trachea, but no significant differences were present between both groups at the respective time points (Fig. 3D–F). The decrease of T cells and macrophages at 14 dpi is in line with the significant reduction of virus antigen at the respective time point (Fig. 2B,C). Immunohistochemistry for detection of CD20^+^ B cells did not reveal any differences between groups and time points in the nasal turbinates. However, at 4 dpi numbers of B cells were significantly increased in the trachea of MVA-S-vaccinated compared to mock-vaccinated animals and compared to the later time point (Fig. 3G–I).

### MERS-CoV has a strong tropism for cytokeratin 18 expressing epithelial cells of the upper respiratory tract

**Figure 4:**
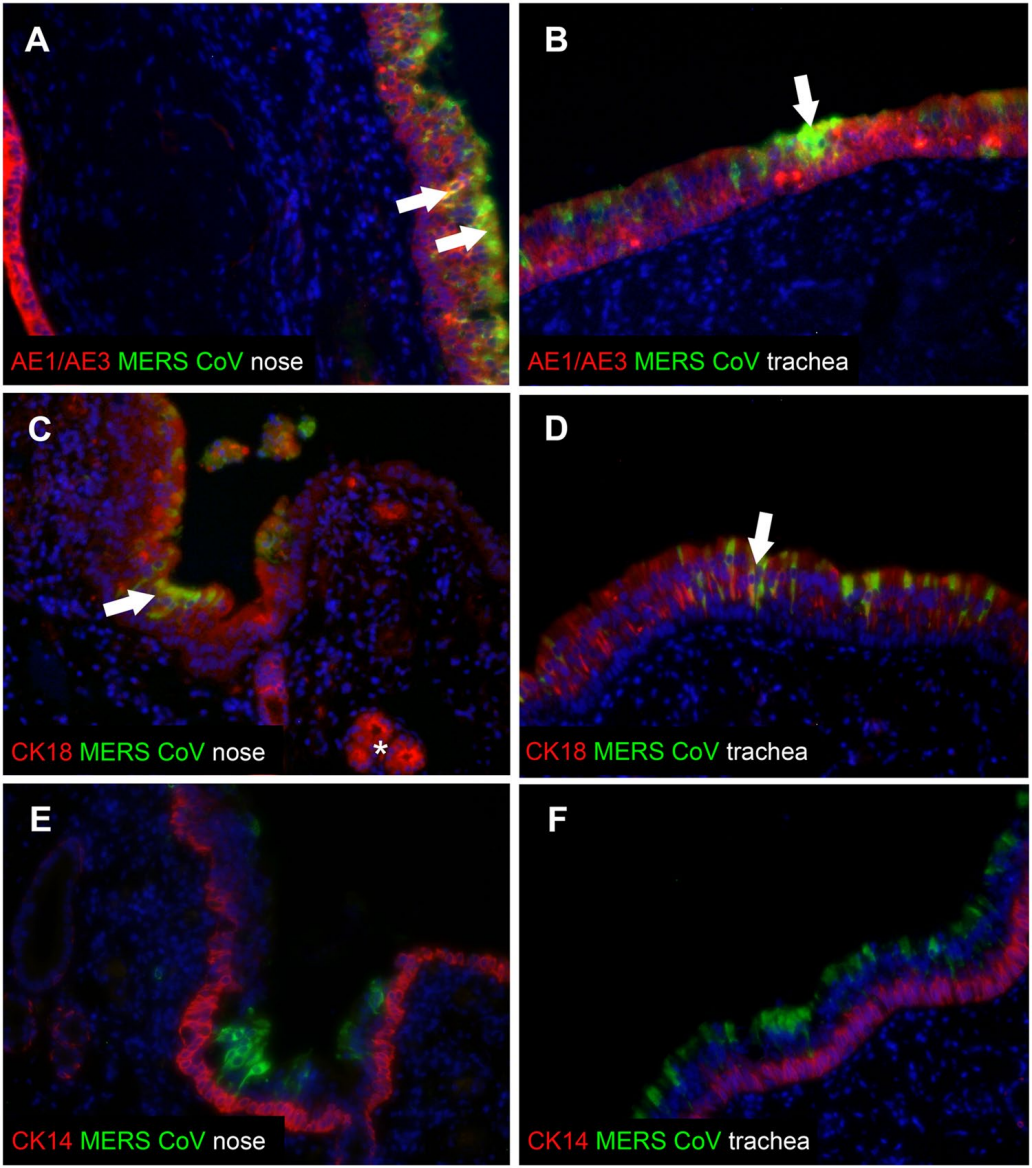
Epithelial tropism of MERS-Coronavirus (MERS-CoV) in experimentally infected, mock-vaccinated dromedaries. (**A**) Visualization of MERS-CoV nucleocapsid antigen (green) and pan-cytokeratin (CK, red) in the respiratory epithelium of nasal turbinates and trachea (**B**). Massive co-localization (yellow, white arrows) in the epithelium of both organs. (**C**) Multifocal co-localization (yellow, white arrow) of CK18 (red) and MERS-CoV nucleocapsid antigen (green) in the respiratory epithelium of nasal turbinates and trachea (**D**). Submucosal glands stain brightly positive for CK18 but lack viral antigen (asterisk in (**C**)). (**E**) No co-localization of CK14 (red) and MERS-CoV nucleocapsid antigen (green) in nasal turbinates and trachea (**F**). A–F: immunofluorescence, 200x.

At 4 dpi, MERS-CoV nucleocapsid antigen was mainly located within the apical epithelial layers of nasal turbinates and trachea of mock-vaccinated animals. For confirmation of these results and elucidation of the cell tropism of MERS-CoV in dromedaries, double immunofluorescence labeling with antibodies specific for MERS-CoV nucleocapsid and pan-CK was performed on these tissues. Double staining revealed massive co-localization of both antigens in the epithelium of nasal turbinates and trachea (Fig. 4A,B). To further distinguish epithelial subsets in the upper respiratory tract tissue of dromedaries, antibodies specific for certain CK of apical and basal cells have been established and were evaluated regarding their distribution (Suppl. Fig. S1). Double immunofluorescence with these antibodies showed strong co-localization of MERS-CoV nucleocapsid with CK18 located in apical epithelial cells at 4 dpi in nasal turbinates and trachea (Fig. 4C,D). There was no co-localization with CK5/6 and CK14, expressed by basal epithelial cells, in any localization (Fig. 4E,F). Moreover, MERS-CoV nucleocapsid antigen was not identified in serous glands, located in the submucosa of the nasal turbinates, even if the apical proportion of these structures stained brightly positive for CK18 (Fig. 4C). At 14 dpi, localization of MERS-CoV nucleocapsid antigen in the nasal turbinates of mock-vaccinated remained basically identical to the distribution at 4 dpi but the number of MERS-CoV positive staining cells was substantially reduced. In MVA-S-vaccinated animals the amount of virus antigen detected by immunofluorescence was very low at 4 dpi, but depicted similar co-localization with CK18.

### Restriction of MERS-CoV infection to apical epithelial cells correlates with the expression of DPP4 by the apical brush border

**Figure 5:**
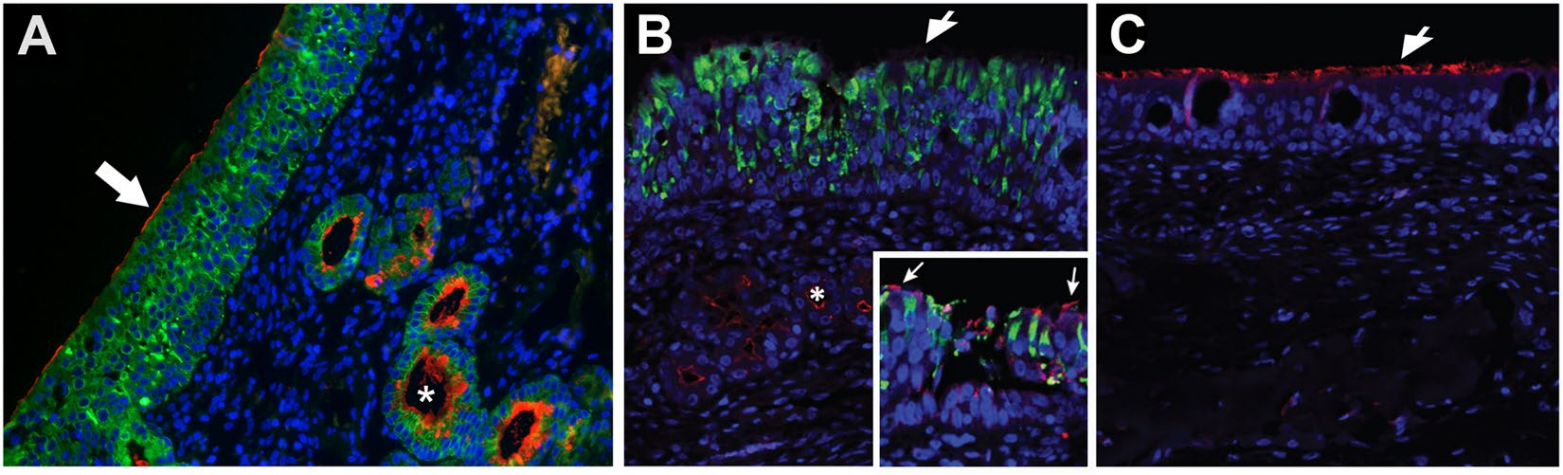
Expression of dipeptidyl peptidase 4 (DPP4) in experimentally infected, mock-vaccinated dromedaries. (**A**) Respiratory epithelium of the nasal turbinates. Double staining of pan-cytokeratin (CK, green) and DPP4 (red). DPP4 is detectable along the apical brush border of CK-positive cells and along the apical surface of submucosal glands (asterisk). (**B**) In MERS-CoV-infected cells of nasal epithelium (green), DPP4 (red) is not demonstrable along the apical surface (arrow) but remains detectable in adjacent cells staining negative for MERS-CoV antigen (insert, white arrows) and in submucosal glandular cells (asterisk). (**C**) In the non-infected nasal epithelium of the same animal, DPP4 (red) is continuously present along the apical surface of ciliated cells (arrow). A–C: Immunofluorescence, 200x.

Double immunofluorescence of the cell surface receptor DPP4 with pan-CK revealed strong expression of DPP4 along the apical brush border of ciliated CK-positive cells and along the apical rim of submucosal gland epithelial cells in MVA-S-vaccinated and mock-vaccinated animals at both investigated time points in nasal turbinates (Fig. 5A) and trachea. Interestingly, DPP4 staining revealed a segmental lack of signal suggestive of multifocal loss of DPP4 expression in MERS-CoV-infected nasal epithelium (Fig. 5B) compared to non-infected nasal epithelium of the same animal in another localization where DPP4 expression remained continuous (Fig. 5C). Loss of DPP4 expression seems to be exclusively restricted to MERS-CoV-infected cells as adjacent cells staining negative for MERS-CoV antigen retained positivity for DPP4 (insert in Fig. 5B). For further characterization, staining of cilia was performed to evaluate whether multifocal lack of DPP4 was associated with concurrent ciliary loss.

### MERS-CoV infection in dromedaries is accompanied by massive ciliary loss and concomitant lack of DPP4 expression by the respiratory epithelium

**Figure 6:**
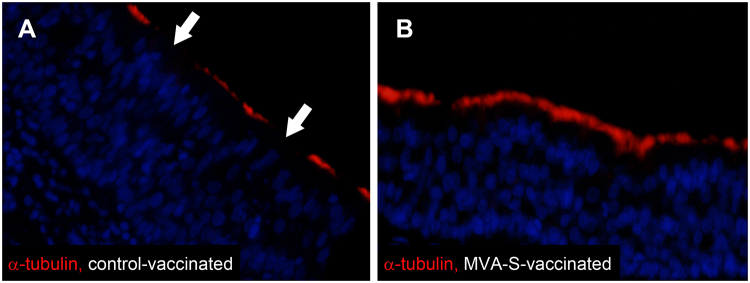
Expression of α-tubulin in MERS-Coronavirus (MERS-CoV)-infected, mock-vaccinated and MVA-S-vaccinated dromedaries. (**A**) Respiratory epithelium of the nasal turbinates. Segmental loss of α-tubulin (red, white arrows) along the apical brush border in a mock-vaccinated animal. (**B**) Respiratory epithelium of the nasal turbinates. Almost uninterrupted expression of α-tubulin (red) along the brush border of a MVA-S-vaccinated dromedary. A-B: immunofluorescence, 200x.

**Figure 7:**
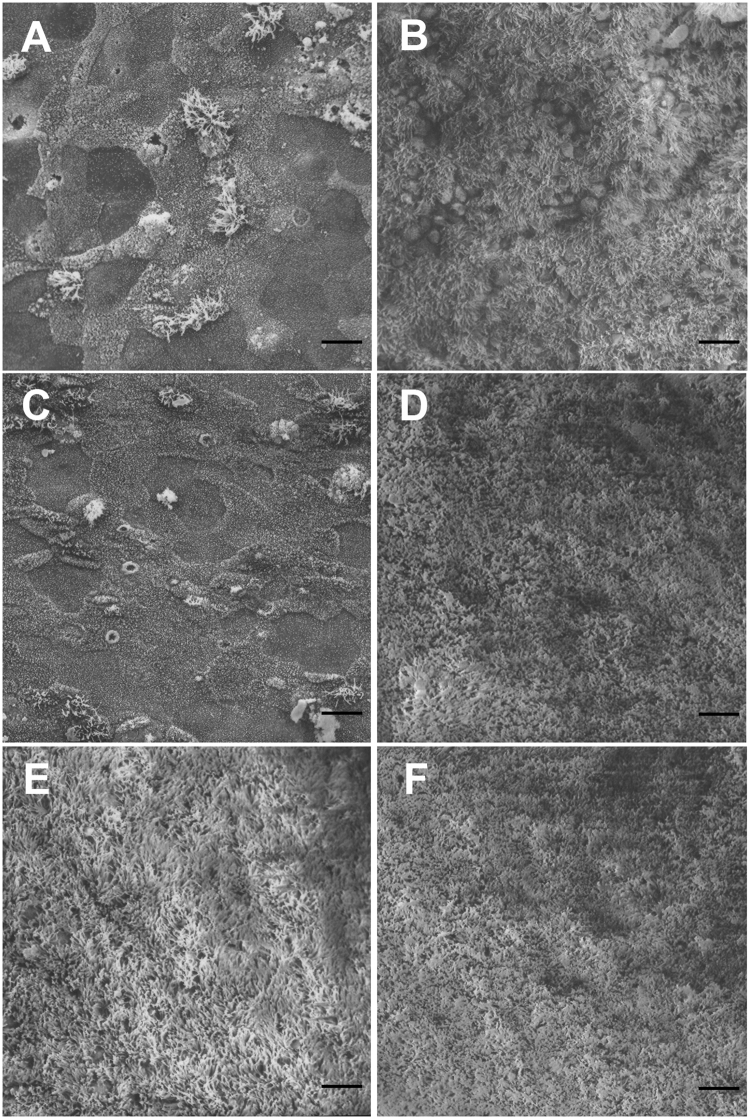
Scanning electron microscopy (SEM) of the upper respiratory tract organs of MERS-Coronavirus (MERS-CoV)-infected, mock-vaccinated and MVA-S-vaccinated dromedaries. (**A**) Respiratory epithelium of the nasal turbinates. Massive ciliary loss in mock-vaccinated animals in comparison to MVA-S-vaccinated animals with regular ciliation (**B**). (**C**) Trachea. Substantial loss of cilia in mock-vaccinated animals compared to MVA-S-vaccinated animals (**D**). (**E**) Bronchus. No differences between mock-vaccinated and MVA-S-vaccinated animals (**F**). A–F: SEM, bar = 10 µm.

Detection of cilia by an anti-α-acetylated tubulin specific antibody in MERS-CoV infected dromedaries revealed a massive and widespread loss of signal in nasal turbinates and trachea of mock-vaccinated animals compared to MVA-S-vaccinated animals at 4 dpi (Figs. 6A,B). As an additional control, nasal tissue of a non-infected control animal was stained with the same antibody and showed a bright and continuous staining without segmental loss as expected. To confirm the massive loss of cilia in infected, mock-vaccinated animals, the respiratory epithelia of nasal turbinates and trachea were additionally investigated by scanning electron microscopy (SEM). SEM showed a widespread and significant loss of cilia in both organs compared to the tissue of the MVA-S-vaccinated animals at 4 dpi (Fig. 7A–F). Since ciliary loss occurred frequently in association with infiltration of inflammatory cells into the epithelium but also in areas with rather mild inflammatory lesions restricted to lamina propria and submucosa, TUNEL assay was performed to determine whether the loss of cilia is directly related to virus-induced apoptosis of epithelial cells. Since the TUNEL assay revealed some but not abundant apoptotic cells in the respiratory epithelium (Suppl. Fig. S2) cilia-specific alterations by MERS-CoV must be assumed.

### MERS-CoV antigen is detectable in macrophages but not in lymphocytes

**Figure 8:**
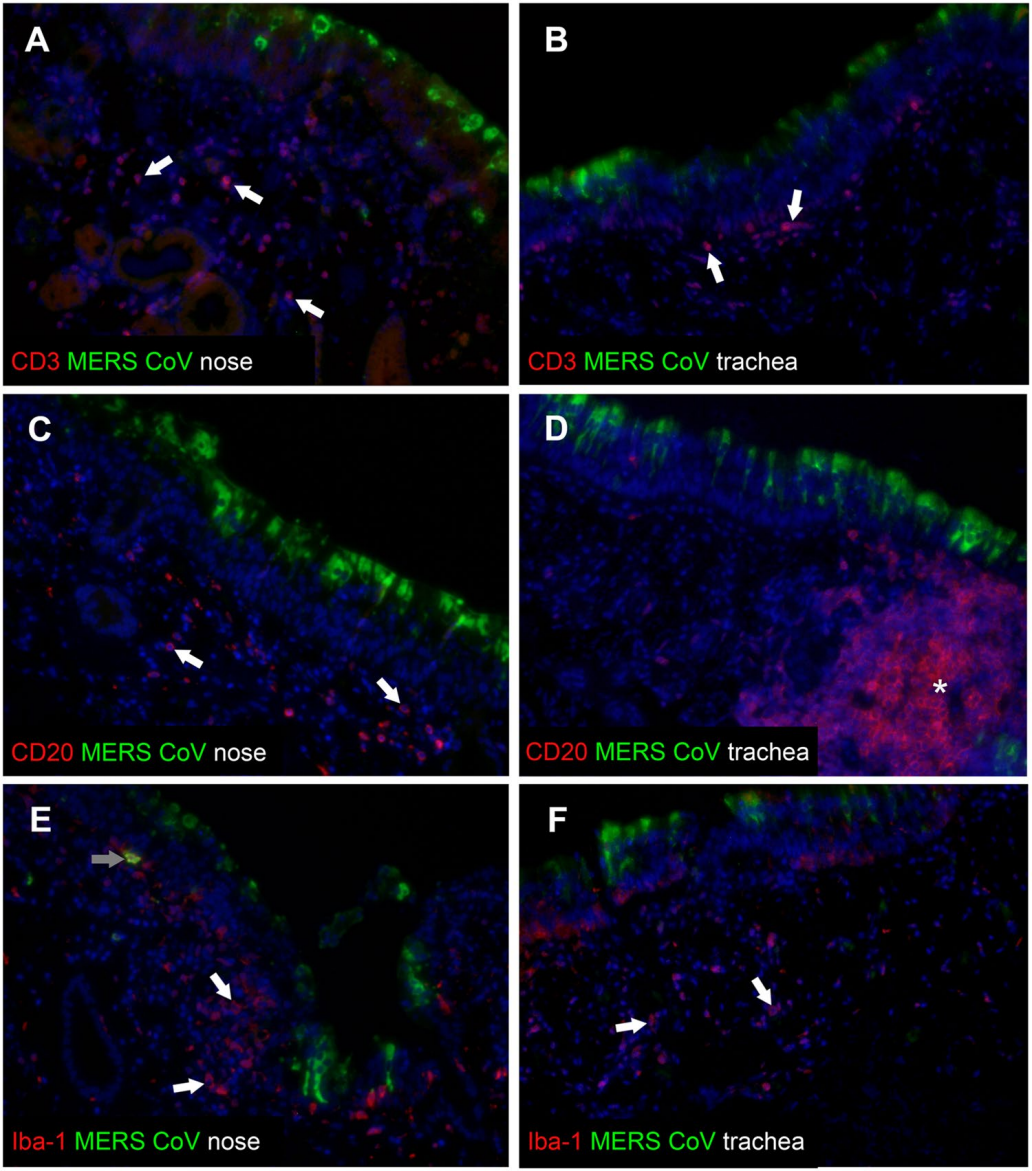
Inflammatory cell tropism of MERS-Coronavirus (MERS-CoV) in experimentally infected, mock-vaccinated dromedaries. (**A**) Visualization of MERS-CoV nucleocapsid antigen (green) and CD3^+^ T cells (red) in the respiratory epithelium of the nasal turbinates and trachea (**B**). T cells stain brightly positive (white arrows) but co-localization with virus antigen was not detected. (**C**) Double staining of MERS-CoV nucleocapsid antigen (green) and CD20^+^ B cells (red) in the respiratory epithelium of the nasal turbinates and trachea (**D**). B cells stain brightly positive (white arrows) and form rarely follicular aggregates (asterisk). Co-localization of both antigens was absent. (**E**) Co-localization (yellow) of MERS-CoV nucleocapsid antigen (green) and Iba-1^+^ macrophages (red) in nasal turbinates and trachea (**F**). Macrophages stain brightly positive (white arrows) and virus antigen was rarely detectable in Iba-1^+^ macrophages in the lamina propria and submucosa of the nasal turbinates (grey arrow in (**E**)). Co-localization of both antigens was absent in the trachea. A–F: immunofluorescence, 200x.

To finally elucidate the histogenesis of cells staining positive for MERS-CoV nucleocapsid antigen within the lamina propria of the nasal turbinates of mock-vaccinated dromedaries, additional double immunofluorescence labeling was performed. Based on histological evaluation of HE stained slides, MERS-CoV antigen was supposed to be located in macrophages or other inflammatory cells and double labeling with antibodies for detection of T cells, B cells, and macrophages was accomplished. Staining of MERS-CoV nucleocapsid antigen with CD3 and CD20 antigen, respectively, did not reveal any co-localization, neither in the nasal turbinates nor in the trachea (Fig. 8A–D). However, single cells within the lamina propria of the nasal turbinates stained simultaneously positive for Iba-1 and MERS-CoV nucleocapsid and were characterized by a macrophage-like morphology (Fig. 8E). In the trachea, double positive staining cell elements were absent (Fig. 8F). For further confirmation the reaction was repeated by use of another macrophage specific antibody. The anti-CD204-specific antibody exhibited an intense co-localization of MERS-CoV nucleocapsid and CD204 in single cells in the same localization. Overall, MERS-CoV nucleocapsid antigen was therefore not only detectable in CK18^+^ apical epithelial cells but also within the cytoplasm of single macrophages. However, co-localization of MERS-CoV nucleocapsid with Iba-1 and CD204, respectively, was not detected within the trachea, implicating that the presence of MERS-CoV in macrophages might be related to high virus loads in the respective localization.

**Figure 9:**
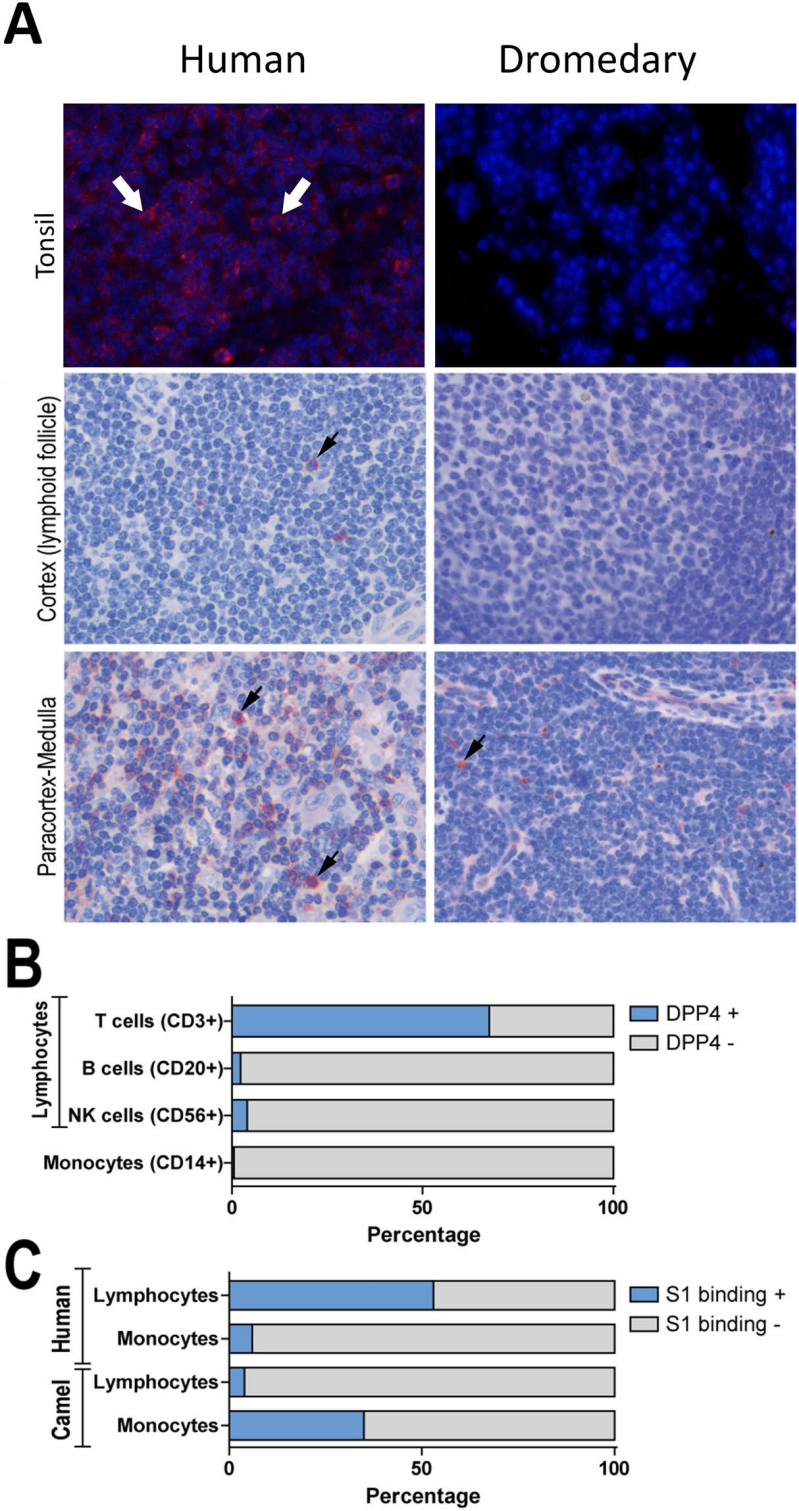
Expression of dipeptidyl peptidase 4 (DPP4) in lymphoid organs of humans and dromedaries. (**A**) DPP4 (red) is frequently detectable on lymphoid cells (white arrows) in the human tonsil but is completely absent in tonsillar tissue of dromedaries. Immunofluorescence, 400x. In the lymph nodes of both human and dromedary, DPP4 (red) is detected in the paracortex and medulla (arrows), but in much smaller numbers in that of dromedaries (arrow). Immunohistochemistry, 400x. In human PBMC, DPP4 is mainly expressed by CD3^+^ T cells and hardly expressed by CD20^+^ B cells, CD56^+^ NK cells, and CD14^+^ monocytes (**B**). S1 protein of MERS-CoV is used to detect DPP4^+^ cells in both human and dromedary PBMC. Lymphocytes and monocytes population are differentiated based on their size and granularity. DPP4 is mainly expressed by lymphocytes in human PBMC while it is mainly expressed by monocytes in dromedary PBMC (**C**). Data in figure B and C are visualized as the mean percentage.

Since DPP4 was only detectable within the apical brush border of the surface epithelium and submucosal glands, but not on the surface of inflammatory cells within lamina propria and submucosa of the nasal turbinates by immunofluorescence, dromedary and human lymphoid tissue were stained for comparison and control. Whereas DPP4 was evident within lymphoid human tonsillar tissue (Fig. 9A), a positive signal was not observed in tonsils of MVA-S-vaccinated and mock-vaccinated dromedaries using immunofluorescence. Similarly, tonsillar tissue of the non-infected control animal did also not reveal any DPP4 expression. In human lymph node tissue DPP4 was rarely detectable by immunohistochemistry on round cells in the cortex and more frequently within the paracortex and medulla. In camels DPP4 was rarely demonstrable within the paracortex and medulla of lymph node but lacked expression in the cortex (Fig. 9A).

In order to elucidate the subset of human immune cells expressing DPP4, flow cytometry was performed and revealed DPP4 expression in human PBMC by CD3^+^ T cells and hardly by CD20^+^ B cells, CD56^+^ NK cells, and CD14^+^ monocytes (Fig. 9B). The S1 protein of MERS-CoV detected DPP4^+^ cells in both human and dromedary PBMC. Lymphocytic and monocytic populations were differentiated based on their size and granularity. DPP4 was mainly expressed by lymphocytes in human PBMC and monocytes in dromedary PBMC even if the expression level was lower in dromedaries than in humans (Fig. 9C).

## Discussion

It has recently been shown that dromedaries play a key role in the transmission of MERS-CoV at the animal-human interface^16^. In addition, experimentally infected dromedaries serve as an important animal model for investigations on certain aspects of MERS-CoV pathogenesis^21,22^. The use of appropriate animal models is highly required, since human tissue samples from individual MERS-CoV cases are rarely accessible^29–31^, which has in part been attributed to cultural reasons^32^. Two individual case descriptions detected viral particles *via* electron microscopy and immunohistochemistry in pneumocytes, pulmonary macrophages, renal proximal tubular epithelial cells, and macrophages within skeletal muscle. Biopsies revealed necrotizing pneumonia, pulmonary alveolar damage, vascular disease, cardiac fibrosis, acute kidney injury, hepatitis, and myositis^30,31^. These reports from human tissue underline that the disease observed in dromedaries after natural and experimental MERS-CoV infection differs substantially from the human counterpart. Whereas dromedaries develop only mild respiratory signs and lack overt pulmonary disease and systemic spread^21,22^, the disease in humans is often accompanied by acute respiratory distress syndrome, renal dysfunction, and lethal outcome^32^. Previous studies indicated that these differences are related to the fact that MERS-CoV predominantly replicates in the lower respiratory tract of humans but not of dromedaries that might, at least in part, be caused by differing expression patterns of the cell surface receptor DPP4. Whereas DPP4 is extensively expressed in the upper respiratory tract epithelia of dromedaries, its expression in the respiratory tract of humans is limited to alveolar epithelial cells and macrophages in the lower airways^25^. In the present study, it has been shown that DPP4 is located on the apical brush border of ciliated CK18 expressing epithelia in the upper respiratory tract of dromedaries. In humans DPP4 can be detected in the brush border of renal proximal convoluted tubules and enterocytes in the intestine^33^ but not within the upper respiratory tract^25^.

The present study demonstrates that acute MERS-CoV infection in dromedaries is accompanied by severe ciliary loss and concomitant lack of DPP4 on infected cells. Adjacent cells in which MERS-CoV antigen is not detectable retain positive staining for DPP4. Ciliary loss and consequent disturbances of mucociliary clearance are a major issue in several viral infections and can foster the development of severe secondary bacterial disease^34^. For instance, common cold in humans is accompanied by a massive loss of cilia and ciliated cells^35^. Similarly, human coronavirus infection of the upper respiratory tract has been described to be associated with migration of axonemes and basal bodies into the cell body (internalization) complemented by loss of cilia on the apical cell surface of infected cells. For the human disease it has been suggested that replicated virions are stored in apical vesicles before they are released. These vesicles may dislocate the basal body and withdraw the cilia into the cell^36^. In dogs, canine respiratory coronavirus infection is also associated with loss and damage to tracheal cilia, accompanied by inflammation^37^. Similar mechanisms might also play a role in MERS-CoV in dromedaries and would at least explain the massive loss of cilia which appears not to be accompanied by massive cell death or other profound histological and ultrastructural alterations in the majority of affected epithelial cells. Interestingly, ciliary loss is accompanied by lack of DPP4, which serves as a cell entry receptor for MERS-CoV in dromedaries. Therefore, the authors suggest that the mild and transient disease in dromedaries is, at least in part, attributable to the downregulation of its own cell entry receptor. Further studies need to be performed to elucidate underlying mechanisms of DPP4 loss in MERS-CoV-infected CK18 positive staining cells of dromedaries. The remaining expression on adjacent MERS-CoV negative cells suggests a potential direct virus mediated mechanism.

The detection of MERS-CoV nucleocapsid antigen in the cytoplasm of a limited number of CD204/Iba-1 positive staining cells within the lamina propria of the nasal turbinates of mock-vaccinated dromedaries at 4 dpi is similar to previous investigations which detected viral antigen and RNA in mononuclear cells and stellate cells of mediastinal lymph nodes in experimentally infected rhesus macaques^9^ and rarely within large mononuclear cells in the tracheal lymph node of infected dromedaries^22^. Moreover, infection of human monocyte-derived dendritic cells and monocyte-derived macrophages has been described *in vitro* and was accompanied by release of viral particles^38,39^. The infection of these cell types is supposed to be mediated by DPP4, expressed on the cell surface of human macrophages^38,40^, and leads to suppression of the innate immunity by reduced expression of tumor necrosis factor (TNF) and interleukin-6 (IL-6)^41^. In contrast, it remains so far uncertain whether the intracytoplasmic detection of MERS-CoV nucleocapsid antigen in dromedary macrophages represents a true productive or abortive infection or whether it is related to phagocytosis of MERS-CoV fragments. Since the number of positive staining macrophages was very low and DPP4 was not detectable on dromedary tonsils and hardly on dromedary lymphocytes and macrophages in lymph nodes it is not unlikely that the intrahistiocytic detection of MERS-CoV nucleocapsid antigen is related to phagocytosis of viral particles or infected cellular components. Nonetheless the staining of the antigen within affected cells was brightly and diffusely distributed in the cytoplasm and the detection of viral antigen in phagosomes would be suspected to rather appear as discrete spots^42,43^. It might therefore been speculated that the viral antigen detection indeed represents virus infection of dromedary macrophages. However, further investigations have to elucidate whether it is a productive or abortive infection.

The lack of detection of viral antigen in dromedary T and B cells is in contrast to the findings in humans where MERS-CoV is capable of infecting T cells derived from peripheral blood and lymphoid organs^44^. On human T cells DPP4 is widely located on the cell surface and serves as a costimulatory molecule which contributes to T cell activation^45^. Contrarily, DPP4 has not been detected on the surface of dromedary lymphocytes in the tonsil and rarely on those located in lymph nodes of the upper respiratory tract in the present study. These results are in line with the lack of MERS-CoV antigen in infiltrated T and B cells within infected organs of dromedaries. In humans, abortive infection of lymphocytes induces apoptosis by activation of both the intrinsic and extrinsic pathway of apoptosis and is accompanied by massive downregulation of DPP4 on the surface of infected T cells^44^. Similarly, a loss of the DPP4 receptor has been detected on the surface of infected epithelial cells in the present study. Taken together, obtained results suggest that the incapacity to invade T cells, alongside other factors, might contribute to the low pathogenicity of MERS-CoV in dromedaries.

In summary, the present study highlights new and important differences between MERS-CoV infection in humans and dromedaries. Most importantly, ciliary loss and reduction of DPP4 expression represent important features of the disease in dromedaries which will deepen our understanding of MERS-CoV. Further investigations need to elucidate the underlying mechanisms of ciliary loss to gain insights into the pathogenesis of this emerging and life-threatening disease. Just recently a novel alphacoronavirus has been detected in Asian house shrews^46^ and future zoonotic transmissions of such novel and well-known coronaviruses will require a profound understanding of their pathogenesis in different host species to achieve better preparedness.

## Materials and Methods

### Animals and tissue sampling

All investigations were performed on archived postmortem tissue, which has been used in a formerly published animal trial^22^ implemented at the Centre for Research into Animal Health (*Centre de Recerca en Sanitat Animal*, CReSA) in Barcelona, Spain. The detailed experimental settings and animal data of the infection and vaccination trial have been published previously^22^. Shortly, eight dromedaries were inoculated intranasally with 10^7^ half-maximal tissue-culture infectious dose (TCID_50_) of MERS-CoV (EMC/2012, passaged on Vero cells) and previously vaccinated/injected with recombinant Modified Vaccinia Virus Ankara (MVA) expressing the full spike protein of MERS-CoV (MVA-S vaccination, n = 4), non-recombinant MVA (mock vaccination, n = 2) and PBS (mock vaccination, n = 2), respectively. On 4 and 14 dpi necropsy was performed on four animals (two MVA-S-vaccinated, one mock-MVA-vaccinated and one PBS injected) and tissues from respiratory (nasal turbinates, trachea, bronchi, lung, pulmonary, and tracheo-bronchial and cervical lymph node) and non-respiratory organs (salivary gland, liver, spleen, kidney, urinary bladder, adrenal gland, heart, pancreas, tonsil, palatum molle, intestine, abomasum, mesenteric lymph node and eyelid) were fixed in 10% neutral-buffered formalin and routinely paraffin-embedded for histology. Serial sections were mounted on coated glass slides (Superfrost Plus®, Menzel Co.) and stained with HE or processed for immunohistochemistry and immunofluorescence, respectively. Prior to embedding tracheal and nasal tissue were decalcified in 10% disodium-ethylenediaminetetraacetate (EDTA, Serva Electrophoresis GmbH) for 48 h.

As an additional control, nasal turbinate of a non-infected dromedary (collected during routine necropsy at the Department of Pathology, University of Veterinary Medicine Hannover, Germany) was used. Euthanasia of the animal was performed due to non-respiratory disease.

Camel peripheral blood mononuclear cells (PBMC) were obtained from naïve animals from a previous experiment^22^. Human PBMC were obtained from 3 healthy blood donors (Sanquin Bloodbank, Rotterdam, The Netherlands). The use of PBMCs for scientific research was approved by the Sanquin Bloodbank after informed consent was obtained from the blood donors.

The human tonsillar tissue which was used for the establishment of the DPP4- and CEACAM5-specific immunohistochemistry was kindly provided by one of the co-authors (IS) and sampled during tonsillectomy. The human bronchiolar lymph nodes paraffin-embedded tissue samples were obtained from the Erasmus MC Tissue Bank. These tissue samples were taken either from healthy donors or from patients with nonmalignant lung tumors. These tissue samples were residual human biomaterials that were collected, stored, and issued by the Erasmus MC Tissue Bank under ISO 15189:2007 standard operating procedures. Use of these materials for research purposes is regulated according to human tissue and medical research: code of conduct for responsible use (2011).

### Light microscopy, immunohistochemistry, immunofluorescence and TUNEL assay

HE stained cross sections of all organs (MVA-S-vaccinated and mock-vaccinated animals, 4 and 14 dpi) were evaluated by light microscopy for the distribution and characterization of lesions induced by MERS-CoV infection. In addition, all organs were screened for the presence of viral antigen by immunohistochemistry using a previously published monoclonal (mc) mouse antibody (Sino Biological Inc.)^22^.

According to the results obtained by light microscopy and MERS-CoV-specific immunohistochemistry, nasal and tracheal tissues were further analyzed with respect to the immune response. Immunohistochemistry was performed for the detection of CD3^+^ T cells (polyclonal rabbit anti-CD3, DakoCytomation GmbH), CD20^+^ B cells (polyclonal rabbit anti-CD20, Thermo Fisher scientific), and CD204^+^ macrophages (monoclonal mouse anti-human macrophage scavenger receptor, human CD204, BioLogo) as summarized in Table 1. The used markers have been previously established for lymphoid dromedary tissue^28^. Additionally, epithelial cells were labeled by application of different antibodies (Table 1) directed against pan-cytokeratin (CK; monoclonal mouse anti-Human Cytokeratin, DakoCytomation GmbH), CK5/6 (monoclonal mouse anti-Cytokeratin 5/6, DakoCytomation GmbH), CK 14 (polyclonal rabbit anti-Keratin 14, Thermo Fisher Scientific) and CK18 (monoclonal mouse anti-Cytokeratin 18, Abcam). CEACAM5 was investigated using a polyclonal rabbit anti-human CEACAM5 antibody (Abcam) and DPP4 was visualized using a polyclonal goat anti-DPP4/CD26-specific antibody (R&D Systems). Briefly, after dewaxing in Roticlear® (Roth C. GmbH & Co. KG) and rehydration in isopropanol and ethanol (96%, Roth C. GmbH & Co. KG), endogenous peroxidase activity was blocked by incubation of sections in 85% ethanol with 0.5% H_2_O_2_ (VWRTM International GmbH) for 30 min at room temperature (RT). Antigen retrieval was performed by incubating the sections in citrate buffer (2.1 g citric acid monohydrate in 1 l distilled water, adjusted with NaOH to pH = 6.0) for 20 min in a microwave (800 W). Subsequently, sections were transferred to Shandon Coverplates^TM^ (Thermo Electron GmbH) and nonspecific binding was blocked by inactivated 20% rabbit (DPP4) or goat serum (all other antibodies) diluted in phosphate buffered saline (PBS) including 1% bovine serum albumin (BSA; PBS/BSA) for 30 minutes. Afterwards sections were incubated with the respective primary antibody for 90 min at RT. For appropriate negative controls, primary antibodies were replaced by ascites fluid from Balb/c mice (1:1000; CD204, pan-CK, CK5/6, CK18, mc MERS-CoV nucleocapsid), goat (1:3000; DPP4) and rabbit serum (1:3000; CD3, CD20, CK14, CEACAM5), respectively. Subsequently, the appropriate secondary biotinylated antibody was added in a dilution of 1:200 with PBS (Table 1). Incubation for 60 min at RT was followed by treatment with the avidin-biotin-peroxidase complex (Vectastain ABC Kit Standard, VectorLaboratories) according to the manufacturer’s protocol. Visualization of the reaction was achieved by the chromogen 3,3-diaminobenzidine tetrahydrochloride (DAB, 0.05%, Sigma Aldrich Chemie GmbH) and addition of 0.03% H_2_O_2_. Slides were finally slightly counterstained with Mayer’s hematoxylin (Roth C. GmbH & Co KG).

**Table 1:** Antigen, clonality, species, source, antigen retrieval, dilution and secondary antibodies used for immunohistochemistry and immunofluorescence.

Antigen	Clonality, Clone, Species	Source	Antigen Retrieval	Immunohistochemsitry		Immunofluoresecence	
				Dilution	Second. Ab	Dilution	Second. Ab
CD3	pc, rabbit	DakoCytomation	Citrate	1:500	GAR-b	1:50	GAR-AlexFlu488
CD20	pc, rabbit	Thermo Fisher scientific	Citrate	1:200	GAR-b	1:50	GAR-AlexFlu488
CD204	mc, SRA-E5, mouse	Transgenic Inc.	Citrate	1:1000	GAM-b	1:20	GAM-Cy^TM^3
Iba-1	pc, rabbit	Wako	Citrate	n.a.	n.a.	1:50	GAR-Cy^TM^3
Pan-CK	mc, AE1/AE3, mouse	DakoCytomation	Citrate	1:500	GAM-b	1:50	GAM-Cy^TM^3
CK5/6	mc, D5/16 B4, mouse	DakoCytomation	Citrate	1:100	GAM-b	1:20	GAM-Cy^TM^3
CK14	pc, rabbit	Thermo Fisher scientific	Citrate	1:500	GAR-b	1:250	GAR-Cy^TM^3
CK18	mc, C-04, mouse	Abcam	Citrate	1:500	GAM-b	1:50	GAM-Cy^TM^3
CEACAM5	pc, rabbit	Abcam	Citrate	1:500	GAR-b	n.a.	n.a.
DPP4	pc, goat	R&D Systems	Citrate	1:50	RAG-b	1:50	DAG-Cy^TM^3
MERS-CoV nucleocapsid	mc, 10, mouse	Sino Biological Inc.	Citrate	1:70	GAM-b	1:70	GAM-Cy^TM^2
MERS-CoV nucleocapsid	pc, rabbit	Sino Biological Inc.	Citrate	n.a.	n.a.	1:2000	GAR-Cy^TM^2
α-acetylated tubulin	mc, 6-11B-1, mouse	Sigma Aldrich	Citrate	n.a.	n.a.	1:250	GAM-Cy^TM^3

Ab, antibody; CD, cluster of differentiation; CEACAM5, carcinoembryonic antigen related cell adhesion molecule 5; CK, cytokeratin; DPP4, Dipeptidylpeptidase 4; GAM-b, goat anti-mouse IgG, biotinylated; GAR, goat anti-rabbit, biotinylated; Iba-1, ionized calcium-binding adapter molecule 1; Immunoglobulin G (IgG); mc, monoclonal; MERS-CoV, Middle East respiratory syndrome coronavirus; n.a., not applied; pc, polyclonal; RAG-b, rabbit anti-goat IgG, biotinylated; RAR, rabbit anti-rat IgG, biotinylated.

Subsequently, double immunofluorescence was implemented to determine the cell tropism of MERS-CoV in dromedaries. For this purpose, the two different MERS-CoV nucleocapsid-specific antibodies were used in combination with antibodies for detection of CD3, CD20, Iba-1 (polyclonal rabbit anti-Iba-1, Wako Chemicals), CD204, CK14, CK18, CK5/6, pan-CK and DPP4, respectively. In addition, single immunofluorescence using anti-acetylated α-tubulin antibody (monoclonal mouse anti-α-tubulin, Sigma-Aldrich) was performed for visualization of the ciliary axoneme^47^. After dewaxing and retrieval of antigens as described above, nonspecific binding was blocked by inactivated 20% horse (DPP4) or goat serum (all other antibodies), respectively, diluted in PBS/0.1% Triton X/1% BSA (PBS/Triton X/BSA) for 30 minutes. Both primary antibodies were applied simultaneously in the respective concentrations (Table 1) diluted with PBS/Triton X/BSA. For appropriate negative controls, primary antibodies were replaced by ascites fluid from Balb/c mice (1:1000; pan-CK, CK5/6, CK18, CD204, mc MERS-CoV nucleocapsid, α-tubulin), rabbit (1:3000; CK14, CD3, CD20, Iba-1, pc MERS-CoV nucleocapsid), and goat serum (1:3000; DPP4), respectively. After incubation over night at 4 °C both secondary antibodies were applied simultaneously (1:200 in PBS/Triton X/BSA, Table 1) and were incubated for 60 min at RT in the dark to visualize the respective antigens. Nuclear counterstaining was performed with 0.01% bisbenzimide (Sigma-Aldrich Chemie GmbH) for 10 minutes and sections were mounted with Dako fluorescence mounting medium (DakoCytomation GmbH).

Terminal deoxynucleotidyl transferase (TdT) dUTP Nick-End Labeling (TUNEL) assay was performed to detect cells that undergo extensive DNA degradation during the late stages of apoptosis on the camel nasal epithelium according to the protocol provided by the manufacturer (Merck KGaA). Tissues pre-treated with DNAse were used as positive control while tissues stained with labelling solution only were used as respective negative control consistent with the manufacturer’s recommendations.

### Flow cytometry

In order to detect which subset of human immune cells expresses DPP4, freshly isolated human PBMCs were incubated with a cocktail of fluorescence labelled monoclonal antibodies, i.e. anti-CD3 (clone SP34-2, BD Biosciences); CD56 (clone MEM188, Serotec); CD20 (clone B9E9, Beckman-Coulter); HLA-DR (clone L243, Biolegend); CD14 (clone M5E2, BD Biosciences); DPP4 (clone 222113, R&D Systems); and live/dead cell viability marker (Thermo Fisher Scientific). Appropriate Ig isotype control antibodies were used as negative controls for each staining. S1 protein of MERS-CoV that recognizes DPP4, used in a previous study^25^, was applied to compare DPP4 expression in camel and human PBMC. Cells were analyzed on a Canto II flow cytometer (BD Biosciences) and using FlowJo® software (FlowJo), then subsequently presented as mean percentage of positive cells.

### Scanning electron microscopy

For scanning electron microscopy, formalin fixed samples of nasal mucosa, trachea, and bronchus of dromedaries infected with MERS-CoV^22^ were post-fixed in 5% glutaraldehyde (Sigma-Aldrich Chemie GmbH) for 24 hours. Afterwards the samples were dehydrated in a series of graded ethanol, dried and coated in a sputter-coater (SCD 040; Oerlikon Balzers) with gold as described previously^48,49^.

### Evaluation, quantification and statistical analysis

For quantification of inflammatory cells and virus by immunohistochemistry five randomly selected images per section, antibody and animal were taken at 400x magnification from the epithelium and adjacent lamina propria and submucosa of nasal turbinates and trachea using an Olympus BX-51 digital camera microscope (Olympus Optical Co. GmbH) and the cell-D imaging software (Olympus Soft imaging Solutions). Afterwards, pictures were evaluated manually by counting immunopositive cells. Statistical analysis was conducted using the Statistical analysis software SAS 9.3 and the Enterprise Guide 5.1 for Windows (SAS Institute Inc.). Pair-wise comparison of groups (MVA-S-vaccinated [n = 10 high power fields] and mock-vaccinated [n = 10 high power fields]) was performed by use of multiple Mann-Whitney-U-Tests. Results were considered statistically significant at *p*-value < 0.05. Column bars were generated with GraphPad Prism 6.0 (GraphPad Software, Inc.).

Slides stained by immunofluorescence were evaluated for co-localization of antigens using an Olympus IX70 inverted fluorescence microscope, the digital camera Olympus DP72 (Olympus Life Science) and the Cell F Imaging Software (Olympus Soft Imaging Solutions GmbH, Olympus Optical Co. GmbH).

For visualization of the results of scanning electron microscopy a digital scanning microscope (DSM 940, Carl Zeiss Jena GmbH) was used. Per localization and time point post infection eight images were taken at 1000x magnification and the percentage of ciliated area was estimated. Data were analyzed using GraphPad Prism 5.0 (GraphPad Software, Inc.). Mann Whitney-U-Test was applied and results were considered statistically significant at *p*-value < 0.05.

## Electronic supplementary material

Supplemental figures
