# Supplementary material for: Experimental infection of dromedaries with Middle East respiratory syndrome-Coronavirus is accompanied by massive ciliary loss and depletion of the cell surface receptor dipeptidyl peptidase 4
Source: Sci Rep. 2018 Jun 27;8:9778. doi: 10.1038/s41598-018-28109-2 (PMC6021449; doi:10.1038/s41598-018-28109-2)
Supplement: Supplementary file 1 — Supplemental figures [file 41598_2018_28109_MOESM1_ESM.pdf]

Experimental infection of dromedaries with Middle East respiratory syndrome-Coronavirus is accompanied by massive ciliary loss and depletion of the cell surface receptor dipeptidyl peptidase 4

Ann-Kathrin Haverkamp[1,#], Annika Lehmbecker[1,2,#], Ingo Spitzbarth[1,2], W. Widagdo[3], Bart L. Haagmans[3], Joaquim Segalés[4,5], Julia Vergara-Alert[4], Albert Bensaid[4], Judith M. A. van den Brand[6], Albert D. M. E. Osterhaus[7], Wolfgang Baumgärtner[1,2,*]

[1]Department of Pathology, University of Veterinary Medicine Hannover Foundation, 30559 Hannover, Germany

[2]Center for Systems Neuroscience, 30559 Hannover, Germany

[3]Department of Viroscience, Erasmus Medical Center, 3015 CN Rotterdam, The Netherlands

[4]IRTA, Centre de Recerca en Sanitat Animal (CReSA, IRTA-UAB), Campus de la Universitat Autònoma de Barcelona, 08193 Bellaterra, Spain

[5]Departament de Sanitat i Anatomia Animals, Facultat de Veterinària, UAB, 08193 Bellaterra, Barcelona, Spain

[6]Department of Pathobiology, Faculty of Veterinary Science, Utrecht University, 3512 JE Utrecht, The Netherlands

[7]Research Center for Emerging Infections and Zoonoses (RIZ), University of Veterinary Medicine Hannover Foundation, 30559 Hannover, Germany

[#]both authors contributed equally

*Corresponding author:

Department of Pathology, University of Veterinary Medicine Hannover Foundation, Bünteweg 17, 30559 Hannover, Germany

Telephone: +49 511 / 953-8621

Fax No.: +49 511 / 953 - 8675

E-mail address: Wolfgang.Baumgaertner@tiho-hannover.de

Supplemental figures

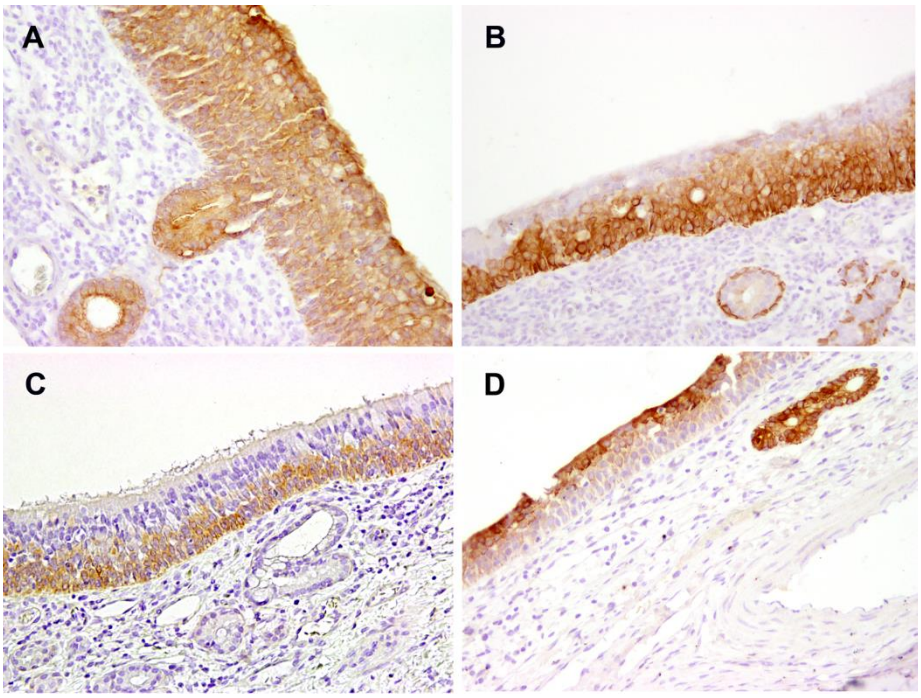

Suppl. Fig. S1. Distribution of different cytokeratins (CK) in the nasal turbinates of dromedaries. (A) Pan-CK stains the entire epithelium including submucosal glands. (B) CK14 is detectable in the basal cell layers of the epithelium and those of submucosal glands. (C) CK5/6 has a similar distribution and is detectable in basal cells but lacks expression in submucosal glands. (D) CK18 stains the apical layers of the epithelium and the apical part of submucosal glands. A: pan-CK-specific immunohistochemistry, B: CK14-specific immunohistochemistry, C: CK5/6-specific immunohistochemistry, D: CK18-specific immunohistochemistry, A-D: 200x.

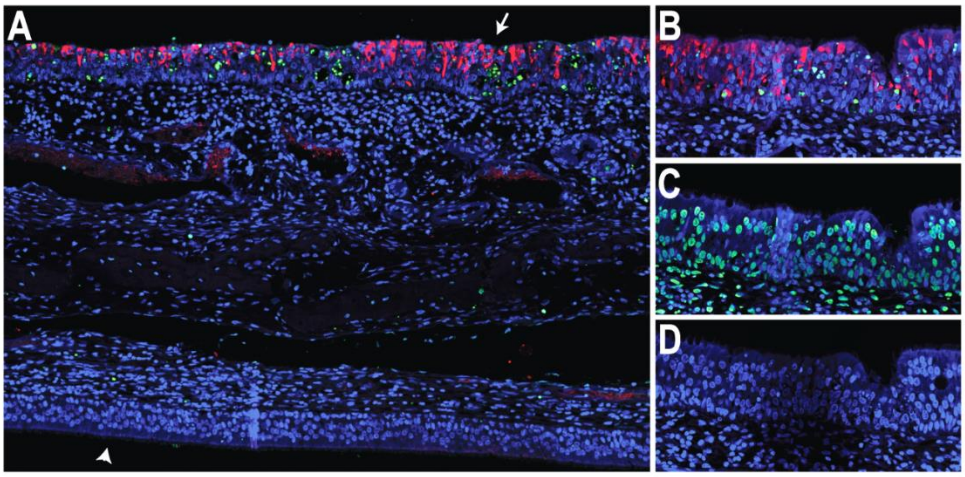

Suppl. Fig. S2. MERS-Coronavirus (MERS-CoV) nucleoprotein (red) and apoptotic cells (green, TUNEL assay) in the nasal epithelium of mock-vaccinated MERS-CoV infected dromedaries. (A) The increase of apoptotic cells is detected in the MERS-CoV-infected nasal epithelium (arrow) in comparison to the non-infected nasal epithelium (arrowhead). (B) Higher magnification of the MERS-CoV-infected nasal epithelium in (A). Co-localization of MERS-CoV nucleoprotein and apoptosis marker is hardly detectable (A, B). (C) Nasal epithelium tissue treated with DNAse is used as positive control for TUNEL assay, while nasal epithelium stained only with labelling solution is used as negative control (D). A: Immunofluorescence, 100x, B-D: immunofluorescence, 400x.
